# A Web-Based COVID-19 Tool for Testing Residents in Retirement Homes: Development Study

**DOI:** 10.2196/45875

**Published:** 2023-11-21

**Authors:** Mansoor Davoodi, Ana Batista, Adam Mertel, Abhishek Senapati, Wildan Abdussalam, Jiri Vyskocil, Giuseppe Barbieri, Kai Fan, Weronika Schlechte-Welnicz, Justin M Calabrese

**Affiliations:** 1 Center for Advanced Systems Understanding Görlitz Germany; 2 Helmholtz-Zentrum Dresden-Rossendorf Dresden Germany; 3 Department of Ecological Modelling UFZ - Helmholtz Centre for Environmental Research Leipzig Germany; 4 Department of Biology University of Maryland College Park, MD United States

**Keywords:** application, COVID-19, optimized testing, pandemic, retirement home, web application

## Abstract

**Background:**

Long-term care facilities have been widely affected by the COVID-19 pandemic. Empirical evidence demonstrated that older people are the most impacted and are at higher risk of mortality after being infected. Regularly testing care facility residents is a practical approach to detecting infections proactively. In many cases, the care staff must perform the tests on the residents while also providing essential care, which in turn causes imbalances in their working time. Once an outbreak occurs, suppressing the spread of the virus in retirement homes (RHs) is challenging because the residents are in contact with each other, and isolation measures cannot be widely enforced. Regular testing strategies, on the other hand, have been shown to effectively prevent outbreaks in RHs. However, high-frequency testing may consume substantial staff working time, which results in a trade-off between the time invested in testing and the time spent providing essential care to residents.

**Objective:**

We developed a web application (Retirement Home Testing Optimizer) to assist RH managers in identifying effective testing schedules for residents. The outcome of the app, called the “testing strategy,” is based on dividing facility residents into groups and then testing no more than 1 group per day.

**Methods:**

We created the web application by incorporating influential factors such as the number of residents and staff, the average rate of contacts, the amount of time spent to test, and constraints on the test interval and size of groups. We developed mixed integer nonlinear programming models for balancing staff workload in long-term care facilities while minimizing the expected detection time of a probable infection inside the facility. Additionally, by leveraging symmetries in the problem, we proposed a fast and efficient local search method to find the optimal solution.

**Results:**

Considering the number of residents and staff and other practical constraints of the facilities, the proposed application computes the optimal trade-off testing strategy and suggests the corresponding grouping and testing schedule for residents. The current version of the application is deployed on the server of the Where2Test project and is accessible on their website. The application is open source, and all contents are offered in English and German. We provide comprehensive instructions and guidelines for easy use and understanding of the application’s functionalities. The application was launched in July 2022, and it is currently being tested in RHs in Saxony, Germany.

**Conclusions:**

Recommended testing strategies by our application are tailored to each RH and the goals set by the managers. We advise the users of the application that the proposed model and approach focus on the expected scenarios, that is, the expected risk of infection, and they do not guarantee the avoidance of worst-case scenarios.

## Introduction

### Overview

The COVID-19 pandemic has significantly impacted long-term care facilities (LCFs) [1-3]. These organizations typically have many older residents who have a high mortality risk after being infected with the SARS-CoV-2 virus. According to the European Centre for Disease Prevention and Control, by May 2020, from 37% to 66% of fatalities due to COVID-19 were found in LCFs [4], specifically 38% in Germany [5]. The case facility rate in pooled data from 6 countries (Belgium, Germany, Ireland, Luxembourg, the Netherlands, and Sweden) reported cases and fatal cases continuously from the fourth quarter (Q4) of 2020 (ie, since national testing capacities and capabilities had stabilized) until Q3 2021. Calculation of the pooled case fatality rate, weighted for the number of reported cases, shows a decline from 23.2% in Q4 2020 to 13.1% in Q1 2021, to 12% in Q2, and a slight increase to 13.8% in Q3 2021. Also, it is reported that at least 23% of COVID-19 deaths in the United States are related to LCFs as of January 2022 [6]. More information on prevalence and mortality in LCFs can be found in Chidambaram [6]. Thus, to prevent the virus from spreading and assure the safety of older adults, the Centers for Disease Control and Prevention and the European Centre for Disease Prevention and Control issued several infection prevention and control recommendations, including social distancing in the facilities, daily screening (testing) of staff and residents, isolation, and visitation restrictions [7,8]. Later during the pandemic, COVID-19 vaccines became available, demonstrating high efficacy in reducing mortality in retirement homes (RHs) but still not perfect for all mutations of COVID-19 [9,10]. However, despite high adherence to the infection prevention and control measures and high vaccination rates, residents are still at risk of contracting the virus.

Controlling the spread of the virus in LCFs such as RHs is a challenge. Due to space limitations, social distancing regulations, and care policies, isolating the residents is often impossible, contributing to the rapid spread of the virus once an infection arrives at the facility. One of the most efficient ways to prevent the virus from spreading is by implementing testing procedures [11,12]. However, performing testing in RHs is not straightforward; usually, the staff in charge of performing the testing also has caring activities and has to dedicate a portion of their work time to testing the residents, which affects the quality of the care services provided. In addition, implementing frequent testing on the residents may cause discontent due to the implications of the testing procedure (ie, discomfort, mobilizations, etc). Therefore, it is crucial to develop optimal testing schedules to balance the time the staff dedicates to testing and testing the residents to minimize the detection time of a probable infection that arrives at the RH. Finding optimal testing schedules manually is not an easy task; managers have to consider a number of interrelated, potentially conflicting factors (eg, staff workload, test duration, and limits on the capacity of tests per day), which is difficult and time-consuming. This challenge can be overcome by using tools that help managers quickly make optimal decisions regarding testing schedules.

In this study, we present a web-based application called the Retirement Home Testing Optimizer (hereafter “the app”). The RHs represent a specific class of organizations. In RHs, there are two main groups that must be considered in our model: (1) the residents, who stay there permanently and cannot be moved to other places even under an emergency situation like a pandemic and (2) the staff, who commute between the RH and their place of residence. Furthermore, due to financial constraints, additional staff cannot be easily hired to conduct testing on the residents. Therefore, the staff has to maintain a balance between time devoted to testing the residents and time spent taking care of them. Due to the continuous presence of residents on-site and circumstances that make strict quarantine within such facilities difficult or impossible, the speed of detection of an incipient outbreak is critical, and testing programs are of paramount importance. Therefore, in contrast to the abovementioned applications, our proposed application is tailored to particular characteristics of LCFs, specifically RHs. The application helps the managers in RHs to efficiently determine testing schedules for the residents that reduce the time to detect infected residents while not overburdening care staff with testing duties, which may lead to a lower quality of services and comfort for the patients. The application was developed considering real data and insights from 5 RHs in Saxony, Germany, and received favorable feedback to be used as a guide tool for developing optimal testing programs. The application is free of charge and available in German and English localizations. We remark that the application is not intended to provide precise recommendations for organizations; thus, it should be used at the users’ discretion.

### Related Applications

There are several COVID-19–related applications and web applications available that focus on preventing the spread of the virus in organizations and indoor environments, but none of them address the introduced problem in RHs. In the following, we briefly review these applications.

Since the beginning of the COVID-19 pandemic, several applications have been launched for different purposes (a review of applications during the COVID-19 pandemic is available in [13,14]). According to Almalki and Giannicchi [13], the purposes of these applications include promoting personal health monitoring of users, raising awareness among people about how to manage exposure to COVID-19 and prevent transmission, and conducting research studies. These applications are offered for mobile devices and include several technical features, with basic health information, contact tracing, symptom tracking, contact alerting, and live statistics being the most common.

The Harvard School of Public Health developed a web-based “COVID-19 Risk Calculator” [15] to reduce the risk of infection by evaluating different control strategies. The tool is based on an aerosol and surface interaction model for infectious diseases [16] that measures the transmission risk in closed environments. The tool considers several features, such as room size, time spent in the room, type of activity, use of protective masks, and personal habits, among others. The application computes transmission risk based on the user’s inputs, categorizing it by the relative transmission contribution. Similarly, the Max Planck Institute for Chemistry developed the web application “COVID-19 Aerosol Transmission Risk Calculator” [17] for estimating the risk of transmission in indoor environments [18]. The application includes environments such as classrooms, offices, receptions, choir rehearsals, and supermarkets. The risk of infection is computed considering a number of features, including the properties of the infectious person, room properties, and event details. The Where2Test research group at the Center for Advanced Systems Understanding developed a web-based workplace occupancy optimizer [19]. The workplace optimizer helps managers make better-informed decisions on office presence during the COVID-19 pandemic. The application considers several input parameters to strike a balance between office occupancy, infection risk, and productivity in the workplace. The users can tailor the results according to the number of employees in the organization, the percentage of vaccinated employees, and the local incidence and vaccination rates.

## Methods

### Overview

The application is part of a web-based platform dedicated to developing tools that help organizations identify optimal testing strategies during the COVID-19 pandemic. The Where2Test platform [20] contains several tools, including a workplace occupancy optimizer, risk calculator, wastewater dashboard [21], and forecast dashboards based on different mathematical and data-driven models. The applications are supported by an underlying operational data store, which provides updated COVID-19–related data from Germany, Poland, and Czechia [22]. The website is available in English and German, with the possibility of extension to other languages. In this section, we present the architecture and the development process of the application.

The application architecture consists of the following three functional components: (1) the mathematical model, (2) the backend application programming interface (API), and (3) the interactive web interface.

In order to develop the mathematical model, we first gathered information from RHs in Saxony, Germany, about the common challenges faced during the pandemic and the main features and influential factors to consider. The information helped to improve the model to balance staff workload while minimizing the expected detection time of a probable infection in RHs. The expected risk of infection is computed by using a probabilistic disease transmission model. The mathematical model and a local search algorithm find optimal testing policy given user input (details about the underlying model used in the application are available in Davoodi et al [23]).

The primary goal of the mathematical model is to mimic the disease transmission mechanism inside the RHs when control strategies such as testing are incorporated. For given parameters, it finds the best testing schedule that balances staff workload while minimizing the expected detection time of a probable infection. The input parameters consider the size of the RH (number of employees and number of residents), as well as the testing possibilities (time needed for a single test and preparation time) and the testing strategy calibration (maximum possible workload of the employees, minimal testing frequency, and the maximal size of the testing group). The model is constructed and later optimized based on the knowledge we gathered directly from communication with 3 distinct RH facilities in Saxony, Germany. We make the assumption of homogeneity in terms of the chances of contracting the virus and spreading it for the sake of simplicity. In fact, we use the average values of these parameters in the model. We further assume that the staff is routinely tested due to stringent regulatory requirements, so we concentrate only on testing the residents. For more details about the model, see Davoodi et al [23].

We use the underlying disease transmission model to build the application, considering a representational state transfer [24], which allows efficient state transfer between the back end and front end of the application.

Last but not least, the application consists of an interactive web interface. This layer allows the users to interact with the model in a graphical environment and effectively communicate the outcomes through predefined texts and visuals. The web interface is created as a client application hosted on the server and accessible through the website [20]. The application is built in the TypeScript language using the libraries *React* [25], *Bulma* [26], and *Konvajs* [27].

All 3 components of the application are versioned inside a GitLab (GitLab Inc) repository. To support automatic tests and deployment capabilities, we use the continuous integration actions defined in .yml files. We operate in three environments: (1) the development version, which runs on a local machine; (2) the staging version, which is the most current version of the code deployed on the secondary server, mostly for testing purposes; and (3) the production version, which should work only with the tested and cleaned code. *Pylint* [28] is further used for the automatic testing and analysis of the Python code; we use *webpack* to manage the client-side code [29].

### Ethical Considerations

This study is based on a theoretical model for spreading the SARS-CoV-2 virus in RHs with homogeneous residents. The model suggests a testing strategy for residents, respecting the workload of staff. There has not been an effect or study on humans, and the managers of RHs as the decision maker can use this result as a guideline to improve efficiency. Therefore, no ethics approval was sought.

## Results

### Design and Development

The web interface of the application has a simple design that allows users to easily configure the input parameters and obtain the recommended testing strategy, including the time schedule and the optimal workload for the staff. The layout of the application consists of several modal windows and 2 main panels: user input and the recommended testing strategy. In the following section, we describe the components of the modules in the application and provide a typical use-case scenario.

### Disclaimer Modal

**Figure 1:**
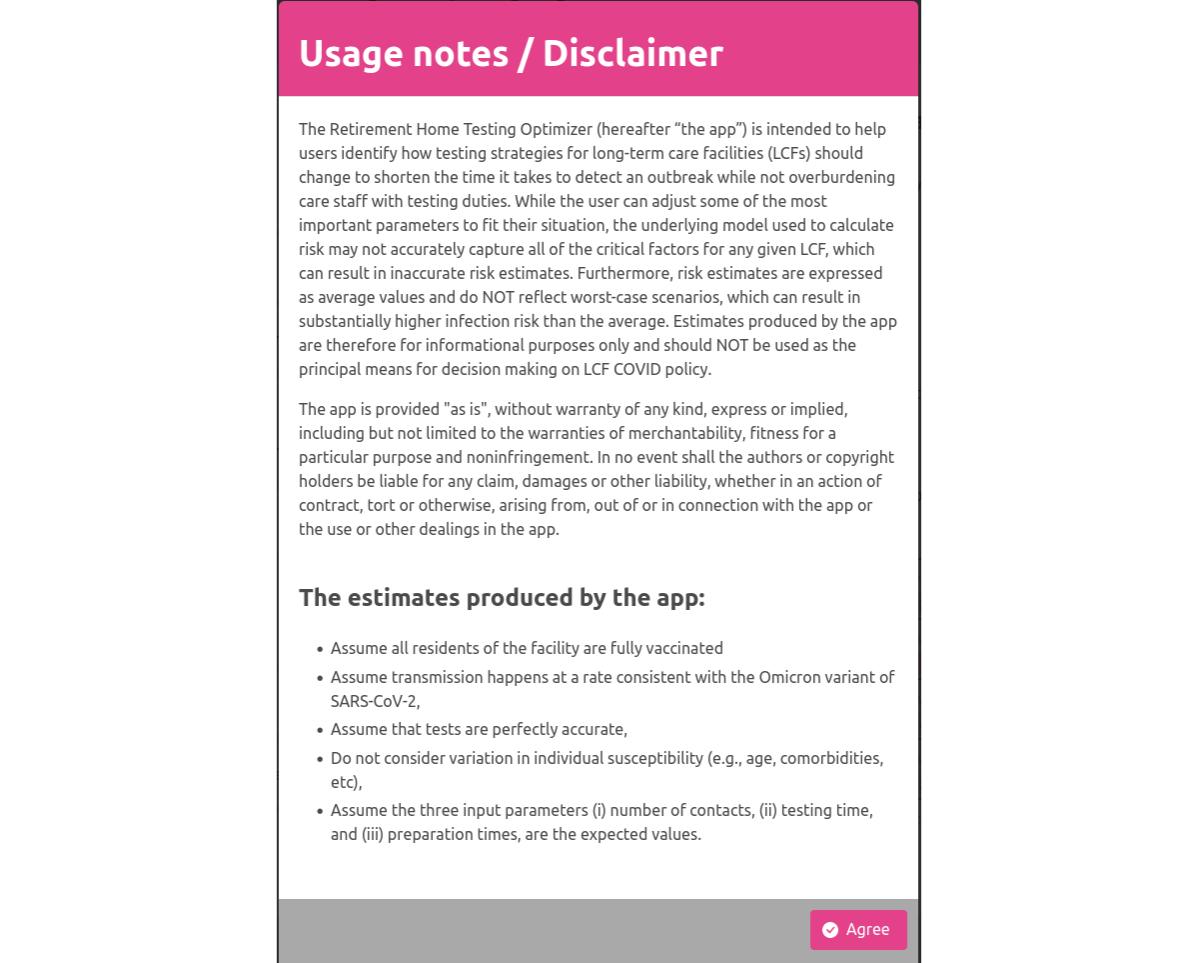
Disclaimer modal informing the users about possible limitations and the underlying assumptions.

The disclaimer modal, as presented in Figure 1, includes use notes about the aims and limitations of the application. The user has to “agree” to acknowledge the limitations to begin using the application. The disclaimer modal also indicates the main assumptions underpinning the application.

### Instructions Modal

The instructions modal includes relevant information for the users about the application’s organization and use. We describe the app’s parameters, output interpretation, limitations, and caveats. This is the place where the user can learn more about the principles of the mathematical model.

### Application Organization and Use

**Figure 2:**
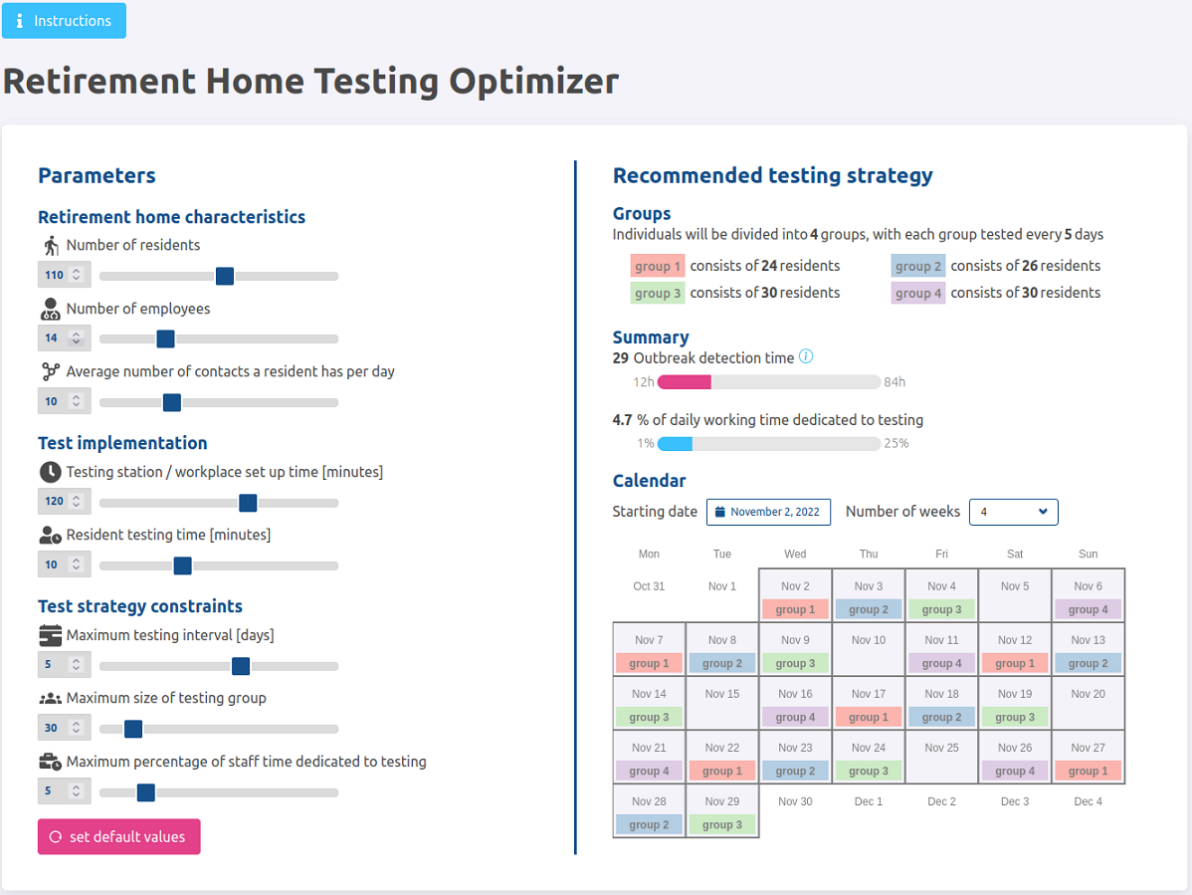
Example of the input parameters (left) and outputs of the app (right).

Each time the user changes any parameter, the client application sends the data to the API layer, which reruns the mathematical model and returns the results. That means the optimization model runs in the background any time one of the inputs is changed, which may take a few seconds to a couple of minutes depending on the input values. During this time, the user sees a progress icon. The application calculates the optimal number of groups, their size (number of residents in each group), and their corresponding testing day. However, it is up to the RH to assign individual residents to testing groups. Next, the application displays the outbreak detection time and staff workload, that is, the percent of workload for the staff that should be assigned to the testing process, associated with the optimal testing strategy. Finally, the application shows the schedule on which the groups should be tested over a time horizon ranging from 3 to 8 weeks (Figure 2 is an example). The following sections describe the content of the panels displayed in the application for user interaction.

#### Left-Hand-Side Panel Description: User Input

The user input panel serves as a form that lists all input parameters. The user can assign each parameter by dragging the slider or manually writing the number into the numeric field element. The application assists the user to determine valid input in two places: (1) each slider and numeric field element is limited by lower and upper bounds that exclude invalid values for the model and (2) in the case of some specific, illogical combination of input values, the application displays a warning text beneath the specific parameter section informing the user about the situation. The amount of time spent on preparation and cleanup activities can be minimized by organizing the residents that will be tested on a given day into a group. In this way, the preparation and cleanup costs are incurred only once for each group, instead of once for each resident. The application requires information on the LCFs’ characteristics and the logistical details of testing within the facility.

The parameters in the left-hand-side panel are divided into 3 blocks: RH characteristics, test implementation, and test strategy constraints. The description of each group of parameters is as follows:

#### RH Characteristics

The parameters under RH characteristics are as follows:

1. Number of residents: the total number of individuals living in the RH. Resident age is not considered.
2. Number of staff: the number of staff who work in the RH as employees and care for the residents. Note that doctors, psychiatrists, or other visitors do not count as staff in this study. The application considers only the shift that performs testing. For instance, in an RH with a total of 100 staff who are scheduled in 3 shifts during the day (eg, 40, 30, and 30 staff members), if the first shift is the only one responsible for testing, then this parameter should be set to 40.
3. Average number of contacts a resident has per day: an estimate of the number of contacts among the residents. This value refers to the average number of interactions that the residents have in common areas and activities during the day. Note that this parameter refers only to the interactions among residents; the contact between the residents and staff is not accounted for.

#### Test Implementation

The parameters under test implementation are as follows:

1. Testing station or workplace setup time (minutes): the total time in minutes for cleaning and preparing the testing workspace.
2. Resident testing time (minutes): the time in minutes dedicated to testing each resident. This value is an estimate of the average time to test a single resident.

#### Test Strategy Constraints

The parameters under test strategy constraints are as follows:

1. Maximum testing interval (days): the maximum allowed time in days between 2 consecutive tests of any given resident. In other words, no resident is allowed to go longer than the maximum testing interval without being tested. The manager decides this value based on the desired frequency to test the residents, the incidence level in the facility’s neighborhood, and regulations.
2. Maximum size of the testing group: the maximum number of residents allowed in any given testing group. The manager decides this value based on logistical considerations.
3. Maximum percentage of staff time dedicated to testing: the aggregate time of staff in 1 working shift dedicated to the testing process. The manager decides the maximum time the staff will dedicate to performing testing on the residents, considering that they have several other tasks during a working shift.

#### Right-Hand-Side Panel Output Description: Recommended Testing Strategy

The right-hand-side panel is dedicated to displaying the outcomes of the mathematical model and communicating the recommended testing strategy to the user. This panel is divided into three sections described as follows:

1. Groups: presents the optimal grouping of residents for the testing. The text of this section informs the user about the number of groups and the number of residents in each.
2. Summary: indicates the average time in hours to the first detection of an outbreak in the RH, as well as the percentage of daily working time dedicated to testing.
3. Calendar: shows the testing program according to the starting date and the number of weeks defined by the user.

### Web Analytics

**Figure 3:**
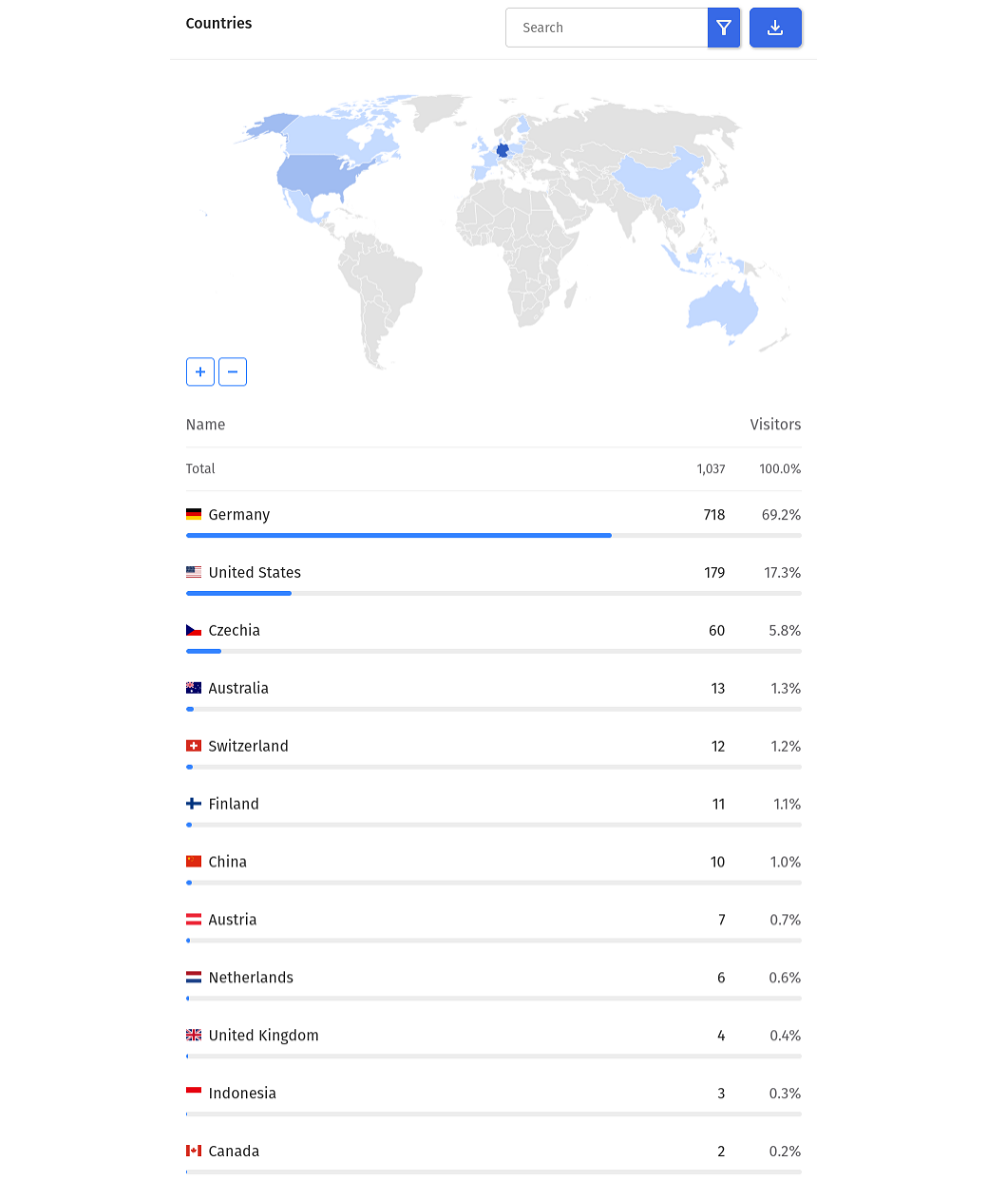
A screenshot from the analytical dashboard microanalytics tool: the geographical distribution of the visitors based on the country of origin.

From the launch of the application on July 1, 2022, to the date of writing this text on November 1, 2022, the Where2Test website was visited by more than 1000 unique visitors (Figure 3 [30]). While we do not track detailed information for every single route, the RH application was the most visited route from all the content on the website. The RH route was also set as a default page for the Where2Test website. We created the analytics report with a tool [30] that tracks the website users with privacy in mind and without using any cookies available on its website.

**Figure 4:**
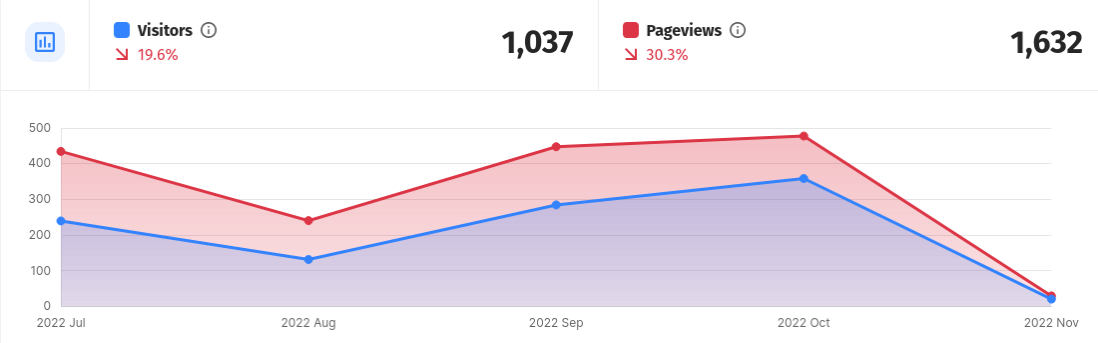
A screenshot from the analytical dashboard of the mictroanalytics tool: the number of unique visitors and page views in time.

From the print screens of the tool’s dashboard (Figure 4 [30]), we can see that most of the traffic came directly from Germany (778/1037, 69.2%), followed by the United States (179/1037, 17.3%) and Czechia (60/1037, 5.8%). The ranking of cities is led by the places in Eastern Germany: Halle (168/1037, 16.2%), Dresden (146/1037, 14.1%), and Görlitz (103/1037, 9.9%). This information may be imprecise, while the visits may be localized based on the internet provider not the actual location of the user. A total of 90% (934/1037) of all users visited the website using a desktop, and only 10% (103/1037) are smartphone users.

## Discussion

### Principal Findings

This paper shows the development process of the web-based application “Retirement Home Testing Optimizer.” The application aims to help RHs define optimal testing schedules during the COVID-19 pandemic. The application was developed considering a mathematical model to balance the time the staff dedicates to testing the residents while minimizing the detection time of a probable infection that arrives at the facility [23]. We used an API that allows efficient state transfer between the back end and front end of the application. The interface of the application is simple to use and is offered in English and German. The users can easily define the input parameters according to the RH characteristics and obtain the results in the output section. The output shows the recommended testing strategy for the residents in RHs, including the grouping of residents for the test, the outbreak detection time, and the percentage of the daily working time dedicated to testing. We also provide a calendar with specific dates to test the residents according to the time frame of evaluation and the starting day defined by the user. The application was validated and tested by an RH in Saxony, Germany, obtaining favorable feedback and opportunities for implementation. We encourage the users to use the results provided by the application carefully, as external circumstances play a major role, including, for example, the frequency of SARS-CoV-2 infections in the region. We advise reviewing the established testing strategy for a particular facility regularly and adjusting it depending on the situation. Therefore, the estimates produced by the application are for informational purposes only and should not be used as the principal means for decision-making on policies in RHs.

The COVID-19 virus spreads mostly through physical contact between people (residents and staff), which is the basis of the model. Therefore, we can assert that the models and the software can be expanded to account for comparable pandemics in the future that spread through direct individual contacts.

### Limitations

We identified several limitations during the development of the web application. First, the application assumes at most 1 round of tests (ie, 1 testing group) per day in the RH and focuses on 1 working shift when computing the testing strategy. However, it can be applied for each shift separately, but the interactions between the shifts and the changing constraints and the contacts among them are not considered. Second, the application includes a set of parameters that managers of RHs must set; some are fixed, while others vary according to the management priorities. The combination of some input parameters may result in an infeasible solution, which indicates that the set of inputs provided is mutually contradictory. To obtain a feasible solution, we suggest the users modify (increasing or decreasing) the values of the test strategy constraints group of parameters because the remaining input parameter groups are generally fixed in the RH. For example, the user might try increasing the maximum group size or the maximum percentage of staff time dedicated to testing. Third, the application computes and finds the optimal testing strategy for most real-size problem instances. However, there may be some input instances of the problem for which the obtained solution is not the exact theoretical optimum but is extremely close to the optimal solution. The expected run time of the application is only a few seconds on average. In some instances (eg, large numbers of residents and staff), the run time may be a couple of minutes in the worst case. Finally, we considered a fixed parameter of the SARS-CoV-2 virus transmission rate, calculated based on the Omicron variant of the virus [31]. However, it can be easily updated in the future as new variants of the virus appear.

### Comparison With Previous Work

In contrast to currently available applications for controlling the spread of the virus in organizations and closed spaces, the application we propose is tailored explicitly to RHs. These facilities have been intensively affected by the COVID-19 pandemic because most residents are at higher risk of mortality after being infected with the virus. The application has been evaluated by RHs in Saxony, Germany, with favorable feedback and potential future implementation. To our knowledge, this is the first web application that can help managers of RHs design an effective COVID-19 testing program without overburdening care staff.

### Conclusions

We developed a web application for RHs in the context of the COVID-19 pandemic. It aimed to minimize the risk of infection and consequent propagation, considering the trade-off between the staff workload allocated to the testing process and the frequency of tests for the residents. Because the residents of RHs have a risk of mortality after infection, any step to shorten detection time is vital and could result in saving lives. The application considerably helps managers to obtain an optimal testing strategy that minimizes the infection risk for any given threshold on the workload. Also, its current version can be further extended to include additional features such as detailed shift scheduling and testing strategies for the staff. Further, the model and the method developed and proposed in this application can be quickly tweaked to fit future probable emerging viral respiratory diseases by considering the underlying disease spread model.

## Abbreviations

API: application programming interface
LCF: long-term care facility
RH: retirement home

## Data Availability

Data sharing is not applicable to this article as no data sets were generated or analyzed during this study. This work presents software (a web-based application) to compute optimal testing strategies in retirement homes. The model and the approach behind the application are explained in Davoodi et al [23], and this paper does not use particular data.
